# A Web-Based Prediction Model for Cancer-Specific Survival of Elderly Patients With Clear Cell Renal Cell Carcinoma: A Population-Based Study

**DOI:** 10.3389/fpubh.2021.833970

**Published:** 2022-03-03

**Authors:** Chenghao Zhanghuang, Jinkui Wang, Zhaoxia Zhang, Liming Jin, Xiaojun Tan, Tao Mi, Jiayan Liu, Mujie Li, Dawei He

**Affiliations:** ^1^Department of Urology, Chongqing Key Laboratory of Children Urogenital Development and Tissue Engineering, Chongqing Key Laboratory of Pediatrics, Ministry of Education Key Laboratory of Child Development and Disorders, National Clinical Research Center for Child Health and Disorders, China International Science and Technology Cooperation Base of Child Development and Critical Disorders, Children's Hospital of Chongqing Medical University, Chongqing, China; ^2^Yunnan Key Laboratory of Children's Major Disease Research, Department of Urology, Kunming Children's Hospital (Children's Hospital Affiliated to Kunming Medical University), Kunming, China; ^3^Department of Urology, Nanchong Central Hospital, The Second Clinical Medical College, North Sichuan Medical University, Nanchong, China

**Keywords:** nomogram, clear cell renal cell carcinoma, cancer-specific survival, elderly patients, SEER

## Abstract

**Background:**

Clear cell renal cell carcinoma (ccRCC) is expected in the elderly and poor prognosis. We aim to explore prognostic factors of ccRCC in the elderly and construct a nomogram to predict cancer-specific survival (CSS) in elderly patients with ccRCC.

**Methods:**

Clinicopathological information for all elderly patients with ccRCC from 2004 to 2018 was downloaded from the Surveillance, Epidemiology, and End Results (SEER) program. All patients were randomly assigned to a training cohort (70%) or a validation cohort (30%). Univariate and multivariate Cox regression models were used to identify the independent risk factors for CSS. A new nomogram was constructed to predict CSS at 1-, 3-, and 5 years in elderly patients with ccRCC based on independent risk factors. Subsequently, we used the consistency index (C-index), calibration curves, and the area under the receiver operating curve (AUC) and decision curve analysis (DCA) to test the prediction accuracy of the model.

**Results:**

A total of 33,509 elderly patients with ccRCC were enrolled. Univariate and multivariate Cox regression analyses results showed that age, sex, race, marriage, tumor size, histological grade, tumor, nodes, and metastases (TNM) stage, and surgery were independent risk factors for CSS in elderly patients with ccRCC. We constructed a nomogram to predict CSS in elderly patients with ccRCC. The C-index of the training cohort and validation cohort was 0.81 (95% CI: 0.802–0.818) and 0.818 (95% CI: 0.806–0.830), respectively. The AUC of the training cohort and validation cohort also suggested that the prediction model had good accuracy. The calibration curve showed that the observed value of the prediction model was highly consistent with the predicted value. DCA showed good clinical application value of the nomogram.

**Conclusion:**

In this study, we explored prognostic factors in elderly patients with ccRCC. We found that age, sex, marriage, TNM stage, surgery, and tumor size were independent risk factors for CSS. We constructed a new nomogram to predict CSS in elderly patients with ccRCC with good accuracy and reliability, providing clinical guidance for patients and physicians.

## Introduction

Renal cell carcinoma (RCC) is a malignant tumor originating from renal tubular epithelial cells. More than 400,000 cases are diagnosed each year, and RCC incidence is about twice as high in men as in women (1, 2). There are several major pathological subtypes of RCC, of which clear cell renal cell carcinoma (ccRCC) accounts for 70–80%, with papillary renal cell carcinoma (PRCC) and chromophobe renal cell carcinoma (CRCC) remaining (3, 4). ccRCC is characterized by rich glycogen and lipids in cells and is manifested as chromosomal 3p deletion and genomic mutation in Von Hippel-Lindau (VHL) tumor suppressor allele (5). Although ccRCC is a disease that can be cured by early surgery, such as radical nephrectomy (RN), partial nephrectomy (PN), and local tumor excision represented by radiofrequency ablation (RFA), recurrence and metastasis occur in up to one-third of cases (6). Moreover, these metastases often suggest poor prognosis, which makes the prognosis of ccRCC significantly different from that of other RCCs (7).

Age is a significant risk factor for cancer. With the increase of age, the probability of cell mutation caused by molecular changes also increases, and cell mutation is generally considered the initiation event of cancer (8). The elderly over 65 years old account for more than 75% of the diagnosed patients with ccRCC. With the aging population and the extension of life expectancy, the incidence rate of renal cancer in the elderly is still increasing year by year (2).

Unfortunately, until today, studies on genetics (9), molecular structure (10), and clinicopathological features (11) have not been able to predict the clinical outcome and prognosis of ccRCC patients accurately. Accurate prediction is crucial in clinical decision-making, patient confidence building, and medical care improvement. In recent years, the application of nomogram prediction models, such as UISS (12), the Stage, Size, Grade, and Necrosis (SSIGN) (13), and Leibovich (14), in the prognosis of cancer patients provides clinicians with a basis for precise and individualized treatment of various cancer patients.

At present, artificial intelligence has been widely used in human health. Awais et al. (15) used texture analysis techniques in fluorescence imaging to help dentists identify areas of oncogenic cavity abnormalities in the oral cavity, and thus, perform biopsies more efficiently to predict the histological diagnosis of epithelial dysplasia. Shah et al. (16) used machine learning to detect the condition and risk of cardiac arrest early to improve survival in patients with heart disease.

However, to our knowledge, no predictive model for cancer-specific survival (CSS) of elderly ccRCC has been reported. Moreover, the prognostic factors of survival in elderly patients with ccRCC remain unclear. We used big data from the Surveillance, Epidemiology, and End Results (SEER) program for the first time to explore factors influencing CSS in elderly patients with ccRCC. Based on the above situation, we developed the nomogram prediction model. We validated its accuracy in evaluating the CSS rate of patients with ccRCC, providing a reference for clinical diagnosis and treatment.

## Patients and Methods

### Data Source and Data Extraction

We downloaded clinicopathological information for all elderly patients with ccRCC from 2004 to 2018 from the SEER program of the National Cancer Institute. SEER database is the National cancer database of the United States, covering about 30% of the population and containing 18 cancer registries. Demographic information, clinicopathological information, and follow-up data for all cancer patients are publicly available from the SEER database. Because patient data are publicly available and cannot be identified, our study needs not require ethical approval or informed consent from patients. Our research methods comply with the regulations of the SEER database.

**Figure 1:**
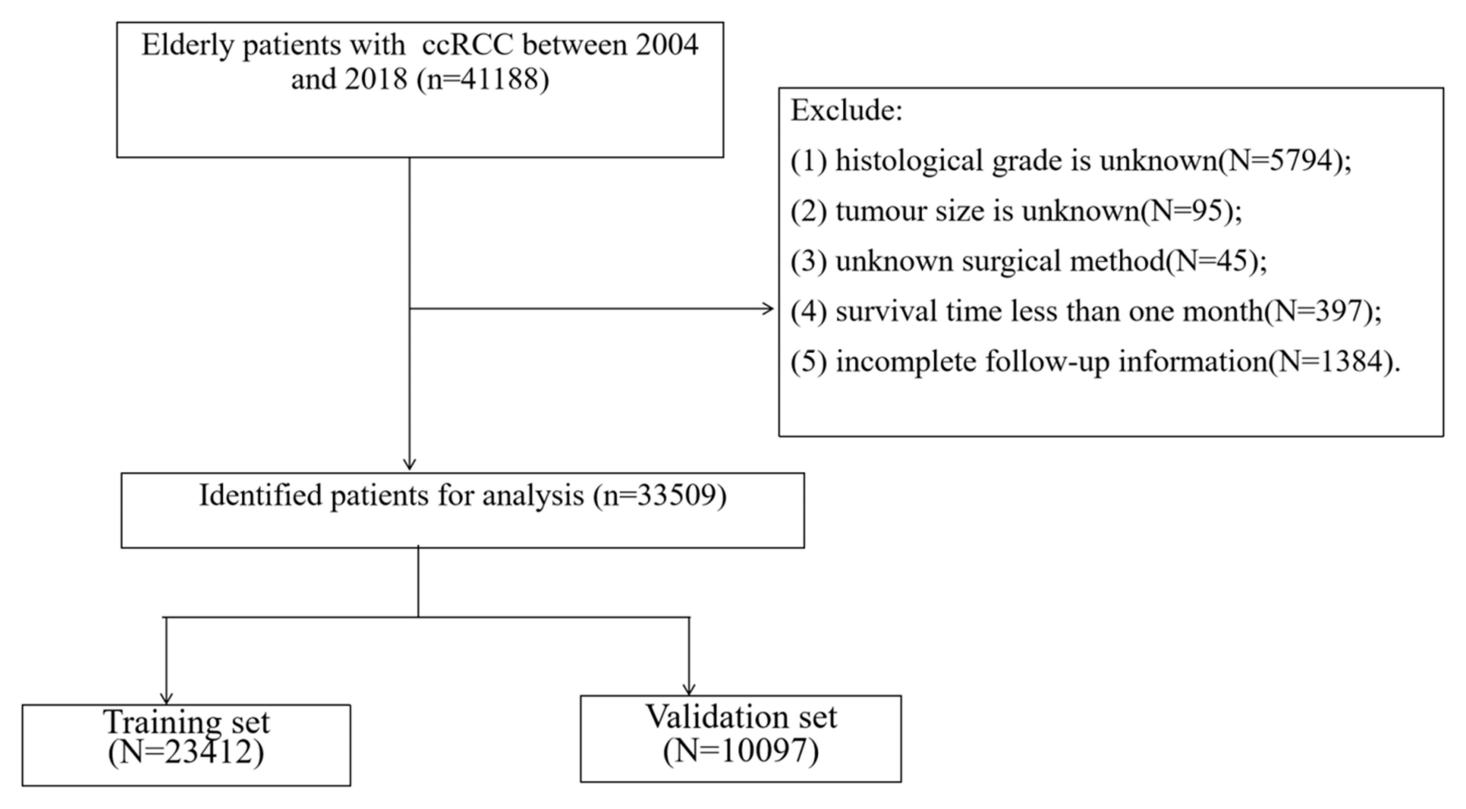
Flowchart for inclusion and exclusion of all patients.

All the demographic information of ccRCC in elderly patients (age, sex, race, year of diagnosis, and marriage), clinical pathologic information (laterality, tumor size, histological grade, and tumor, nodes, and metastases [TNM] stage), treatment (surgery, radiation therapy, and chemotherapy), follow-up information (survival status, survival time, and the cause of death) were collected. Inclusion criteria were as follows: (1) pathological diagnosis of ccRCC (International Classification of Diseases for Oncology [ICD-O]-3 codes, 8310); (2) age ≥65; (3) the years of diagnosis were 2004–2018; and (4) unilateral renal tumor. Exclusion criteria were as follows:(1) tumor histological grade is unknown; (2) tumor size is unknown; (3) unknown surgical method; (4) survival time <1 month; and (5) incomplete follow-up information. The patient screening flow chart is shown in Figure 1.

The patients' race was divided into three categories: white, black, and others (American Indian/AK Native, Asian/Pacific Islander). Patients were divided into years of diagnosis from 2004 to 2010 and 2011 to 2018. The patient's tumor histological grades included grade I (well-differentiated), grade II (moderately differentiated), grade III (poorly differentiated), and grade IV (undifferentiated). Patients were classified as non-operative (SEER Operation Code 0), local tumor excision (SEER Operation Code 10-27), PN (SEER Operation Code 30), and RN (SEER Operation Code 40-80).

### Univariate and Multivariate Cox Regression Analyses

All elderly patients with ccRCC were randomly assigned to a training cohort (70%) or a validation cohort (30%). All variables that might be associated with patient outcomes were included in the analysis. Univariate Cox regression models were used in the training cohort to analyze risk factors associated with in patients with CSS. According to univariate Cox regression analysis, a multivariate Cox regression model was established to identify the independent risk factors for CSS.

### Construction of Nomogram

The establishment of the nomogram mainly relies on the multivariate Cox regression model. We developed a new nomogram to predict CSS at 1-, 3-, and 5 years in elderly patients with ccRCC based on independent risk factors for univariate and multivariate screening. Because these independent risk factors are predictors of patient outcomes.

### Validation of Nomogram

Subsequently, we used the consistency index (C-index) to test the accuracy and discrimination of the prediction model in the training cohort and validation cohort. Calibration curves of 1,000 bootstrap samples were used to test the accuracy of the prediction model. The area under the receiver operating curve (AUC) was used to test the model's prediction accuracy at 1-, 3-, and 5 years.

### Clinical Utility

We used the decision analysis curve (DCA) to validate the clinical potential of the predictive model. DCA is a new algorithm for calculating net benefit under different thresholds and is currently most commonly used to evaluate the clinical application value of models. In addition, we divided patients into high-risk and low-risk groups based on each patient's nomogram score and cutoff values determined by the receiver operating curve. Log-rank test and Kaplan-Meier (K-M) curve were used to compare the survival difference between the two groups. In addition, we analyzed surgical differences between high-risk and low-risk patients based on risk grouping.

### Statistical Analysis

Continuous measurement data were described by means and variance, and comparison between groups was performed by Chi-square test or non-parametric *U* test. Count data were characterized by frequency (%), and a chi-square test was used to compare groups. Univariate and multivariate Cox proportional regression models analyzed the survival and prognostic factors. Log-rank test and Kaplan-Meier (K-M) curve were used to analyze the survival differences between the patients. SPSS 26.0 and R Software 4.1.0 were used for all statistical analyses. Values of *p* < 0.05 were considered statistically significant.

## Results

### Clinical Features

A total of 33,509 elderly patients with ccRCC were enrolled. All patients were randomly divided into the training cohort (N = 23,412) and the validation cohort (N = 10,097). The mean age of all patients was 72.7 ± 5.91 years, 29,034 (86.6%) white, 20,201 (60.3%) men, and 21,239 (63.4%) married. Among all patients, there were 16,447 (49.1%) tumors on the left side, 4,077 (12.2%) tumors at grade I, 17,480 (52.2%) tumors at grade II, 9,595 (28.6%) tumors at grade III, and 2,357 (7.03%) tumors at grade IV. The mean tumor size was 51.8 ± 32.3 mm, the T stage T1a had 14,019 (41.8%), T1b had 8,063 (24.1%), T2 had 2,987 (8.91%), T3 had 8,370 (25.0%), and T4 had 70 (0.21%). There were 32,620 (97.3%) patients in stage N0 and 31,320 (93.5%) patients in stage M0. There were 1,227 (3.66%) patients without surgery, 1,718 (5.13%) patients with local tumor excision, 8,791 (26.2%) patients with PN, and 21,773 (65.0%) patients with RN. There were 1,505 (4.49%) patients who received chemotherapy and 640 (1.91%) patients who received radiotherapy. The clinicopathological information of the patients is shown in Table 1, and there was no significant difference between the training cohort and the validation cohort.

**Table 1:** Clinicopathological characteristics of elderly patients with ccRCC.

	**All**	**Training**	**validation**	
		**cohort**	**cohort**	
	***N* = 33,509**	***N* = 23,412**	***N* = 10,097**	***p***
Age	72.7 (5.91)	72.7 (5.90)	72.8 (5.95)	0.515
Race				0.073
white	29,034 (86.6%)	20,233 (86.4%)	8,801 (87.2%)	
black	2,030 (6.06%)	1,463 (6.25%)	567 (5.62%)	
other	2,445 (7.30%)	1,716 (7.33%)	729 (7.22%)	
Sex				0.313
Male	20,201 (60.3%)	14,156 (60.5%)	6,045 (59.9%)	
Female	13,308 (39.7%)	9,256 (39.5%)	4,052 (40.1%)	
Marriage				0.332
No	12,270 (36.6%)	8,533 (36.4%)	3,737 (37.0%)	
Married	21,239 (63.4%)	14,879 (63.6%)	6,360 (63.0%)	
Year of diagnosis				0.651
2004–2010	13,295 (39.7%)	9,308 (39.8%)	3,987 (39.5%)	
2010–2018	20,214 (60.3%)	14,104 (60.2%)	6,110 (60.5%)	
Laterality				0.138
Left	16,447 (49.1%)	11,554 (49.4%)	4,893 (48.5%)	
Right	17,062 (50.9%)	11,858 (50.6%)	5,204 (51.5%)	
Grade				0.760
I	4,077 (12.2%)	2,834 (12.1%)	1,243 (12.3%)	
II	17,480 (52.2%)	12,212 (52.2%)	5,268 (52.2%)	
III	9,595 (28.6%)	6,698 (28.6%)	2,897 (28.7%)	
IV	2,357 (7.03%)	1,668 (7.12%)	689 (6.82%)	
T				0.266
T1a	14,019 (41.8%)	9,743 (41.6%)	4,276 (42.3%)	
T1b	8,063 (24.1%)	5,694 (24.3%)	2,369 (23.5%)	
T2	2,987 (8.91%)	2,113 (9.03%)	874 (8.66%)	
T3	8,370 (25.0%)	5,811 (24.8%)	2,559 (25.3%)	
T4	70 (0.21%)	51 (0.22%)	19 (0.19%)	
N				0.080
N0	32,620 (97.3%)	22,815 (97.5%)	9,805 (97.1%)	
N1	889 (2.65%)	597 (2.55%)	292 (2.89%)	
M				0.362
M0	31,320 (93.5%)	21,902 (93.6%)	9,418 (93.3%)	
M1	2,189 (6.53%)	1,510 (6.45%)	679 (6.72%)	
Tumor size	51.8 (32.3)	51.7 (31.0)	52.0 (35.0)	0.449
Surgery				0.426
No	1,227 (3.66%)	867 (3.70%)	360 (3.57%)	
Local tumor excision	1,718 (5.13%)	1,173 (5.01%)	545 (5.40%)	
Partial nephrectomy	8,791 (26.2%)	6,127 (26.2%)	2,664 (26.4%)	
Radical nephrectomy	21,773 (65.0%)	15,245 (65.1%)	6,528 (64.7%)	
Chemotherapy				0.038
No/Unknown	32,004 (95.5%)	22,397 (95.7%)	9,607 (95.1%)	
Yes	1,505 (4.49%)	1,015 (4.34%)	490 (4.85%)	
Radiation:				0.354
No/Unknown	32,869 (98.1%)	22,976 (98.1%)	9,893 (98.0%)	
Yes	640 (1.91%)	436 (1.86%)	204 (2.02%)	

### Univariate and Multivariate Cox Regression Analyses

Univariate Cox regression model was used to analyze all variables to screen for variables associated with CSS in elderly patients with ccRCC. Age, sex, race, marriage, year of diagnosis, tumor size, histological grade, TNM stage, and surgery were prognostic factors related to patient survival. We included these factors in a multivariate Cox regression analysis. We found that age, sex, race, marriage, tumor size, histological grade, TNM stage, and surgery were independent risk factors for CSS in elderly patients with ccRCC. In other words, these independent risk factors can be used as factors in predictive models to predict CSS of the patients. The univariate and multivariate analyses results are shown in Table 2.

**Table 2:** Univariate and multivariate analyses of CSS in training cohort.

	**Univariate**			**Multivariate**		
	**HR**	**95%CI**	**P**	**HR**	**95%CI**	**P**
Age	1.04	1.03–1.04	<0.001	1.057	1.053–1.06	<0.001
Race						
white	Reference			Reference		
black	0.83	0.72–0.96	0.013	1.129	1.035–1.232	0.006
other	0.91	0.8–1.03	0.137	0.879	0.806–0.959	0.004
Sex						
Male	Reference			Reference		
Female	0.81	0.76–0.87	<0.001	0.834	0.796–0.874	<0.001
Marriage						
No	Reference			Reference		
Married	0.92	0.86–0.98	0.01	0.852	0.814–0.893	<0.001
Year of diagnosis						
2004–2010	reference					
2010–2018	0.91	0.85–0.97	0.006			
Laterality						
Left	Reference					
Right	1.03	0.97–1.1	0.32			
Grade						
I	Reference			Reference		
II	1.32	1.15–1.5	<0.001	1.037	0.965–1.113	0.32
III	2.7	2.36–3.08	<0.001	1.207	1.117–1.305	<0.001
IV	7.07	6.12–8.17	<0.001	1.797	1.626–1.987	<0.001
T						
T1a	Reference			Reference		
T1b	2.14	1.93–2.38	<0.001	1.176	1.105–1.251	<0.001
T2	4.56	4.06–5.11	<0.001	1.189	1.088–1.299	<0.001
T3	6.19	5.65–6.78	<0.001	1.388	1.294–1.488	<0.001
T4	26.49	16.75–41.89	<0.001	1.866	1.178–2.955	0.008
N						
N0	Reference			Reference		
N1	10.3	9.26–11.46	<0.001	2.023	1.82–2.249	<0.001
M						
M0	Reference			Reference		
M1	11.01	10.2–11.87	<0.001	3.049	2.83–3.285	<0.001
Tumor size	1.01	1.01–1.01	<0.001	1.003	1.002–1.004	<0.001
Surgery						
No	Reference			referEnce		
Local tumor excision	0.1	0.08–0.13	<0.001	0.421	0.368–0.481	<0.001
Partial nephrectomy	0.08	0.07–0.09	<0.001	0.282	0.253–0.314	<0.001
Radical nephrectomy	0.25	0.22–0.27	<0.001	0.361	0.329–0.396	<0.001

### Nomogram Construction for 1-, 3-, and 5-Year CSS

**Figure 2:**
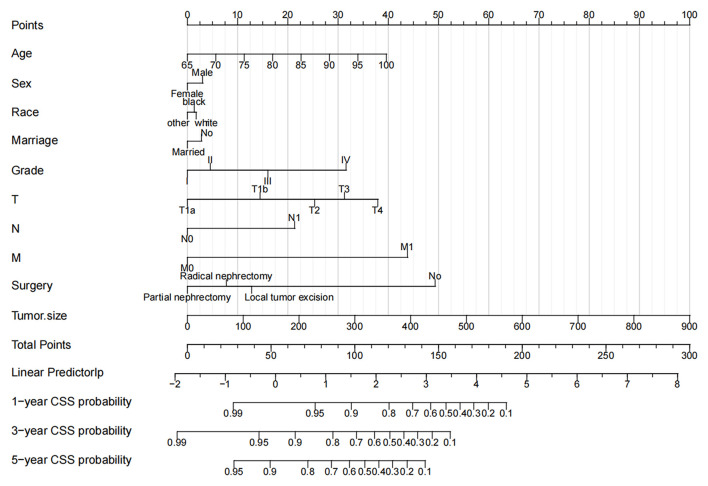
The nomogram predicts CSS in elderly patients with ccRCC at 1-, 3-, and 5 years. CSS, cancer-specific survival; ccRCC, clear cell renal cell carcinoma.

We constructed a nomogram to predict CSS in elderly patients with ccRCC (Figure 2). Tumor size was the most significant risk factor for in patients with CSS, as shown in the nomogram. The larger the tumor, the higher the patient's risk of death. Second, surgery is the second most important factor affecting patient survival. Survival was most increased in patients with PN, followed by RN and local tumor excision and was lowest in patients who did not undergo surgery. Tumor TNM stage and histological tumor grade are important risk factors for patient prognosis. Patients with higher TNM stage or histological grade had lower survival rates. In addition, the age of the patients was also a key factor; the older the patient, the greater the risk of death. Finally, married patients, women, and other races had a relatively high survival rate.

### Validation of the Nomogram

**Figure 3:**
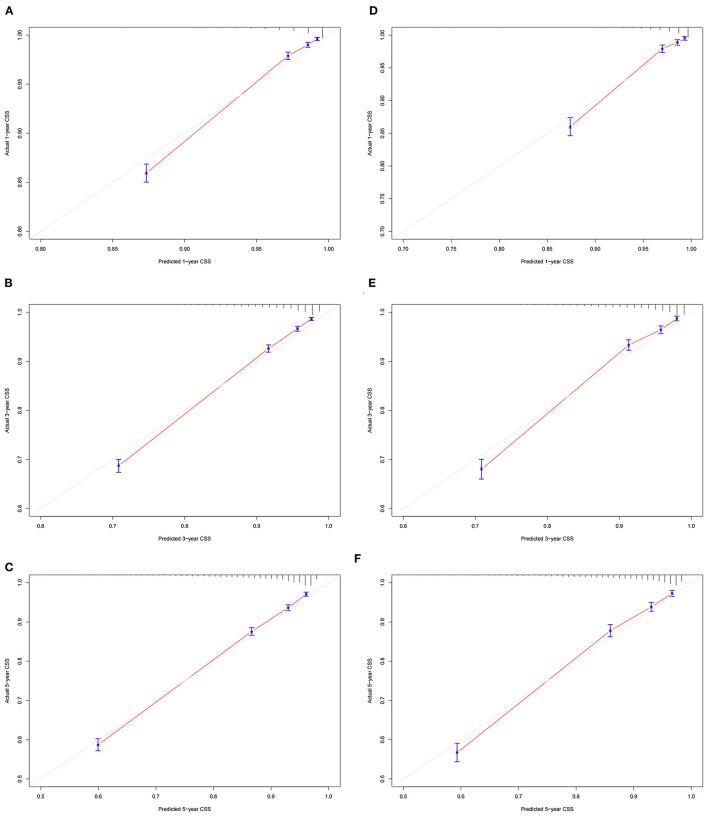
Calibration curve of the nomogram. **(A–C)** Calibration curves of 1 -, 3 -, and 5 years CSS in the training cohort; **(D–F)** calibration curves of 1-, 3-, and 5 years CSS in the validation cohort. CSS, cancer-specific survival.

**Figure 4:**
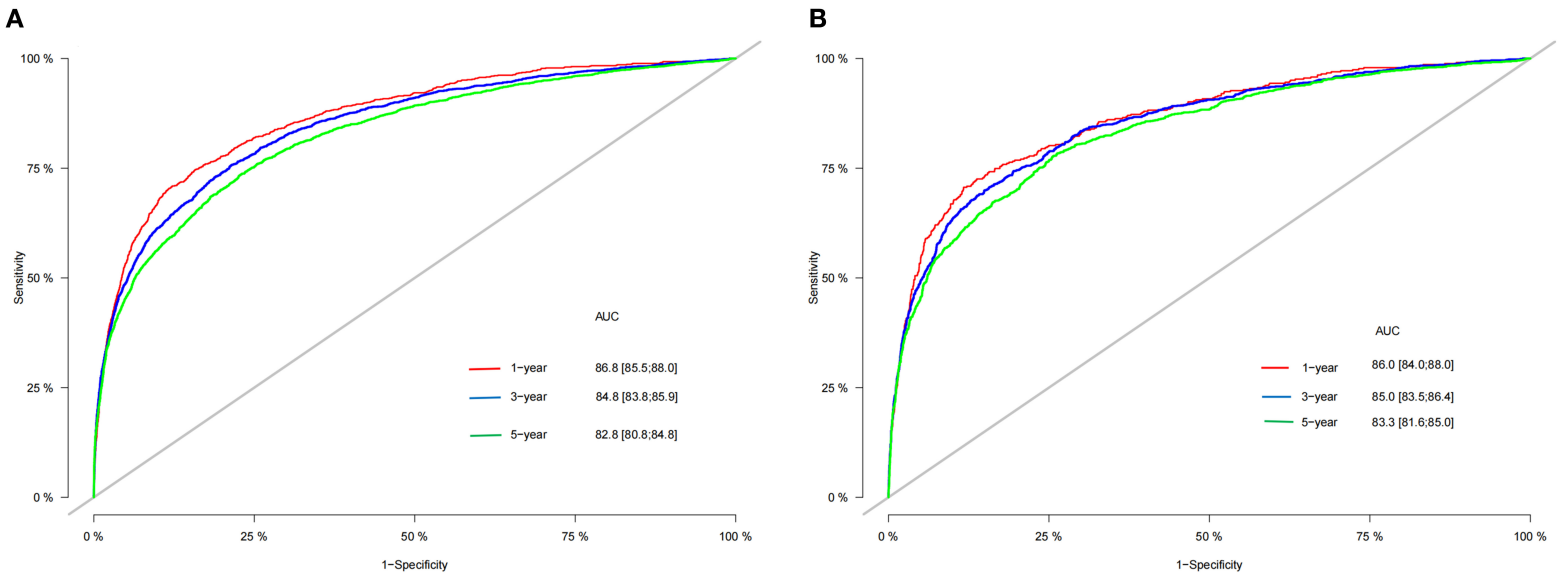
AUC for predicting 1-, 3-, and 5 years CSS in training cohort **(A)** and validation cohort **(B)**. CSS, cancer-specific survival; AUC, area under the receiver operator curve.

In the training cohort and validation cohort, we first use the C-index to validate the accuracy of the nomogram. The C-index of the training cohort and validation cohort was 0.81 (95% CI: 0.802–0.818) and 0.818 (95% CI: 0.806–0.830), respectively, indicating the accuracy and discrimination of the nomogram. In the training cohort and validation cohort, the calibration curve showed that the observed value of the prediction model was highly consistent with the predicted value, proving that the prediction model had good accuracy (Figure 3). The AUC of the training cohort and validation cohort also suggested that the prediction model had good accuracy (Figure 4). In the training cohort, the AUC of the prediction model was 86.8 (95% CI, 85.5–88.0), 84.8 (95% CI, 83.8–85.9), and 82.8 (95% CI, 80.8–84.8) in 1-, 3-, and 5 years, respectively. In the validation cohort, the AUC of the prediction model was 86.0 (95% CI, 84.0–88.0), 85.0 (95% CI, 83.5–86.4), and 83.3 (95% CI, 81.6–85.0), respectively.

### Clinical Application of the Nomogram

**Figure 5:**
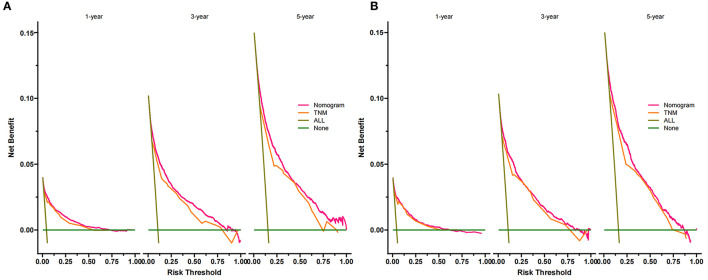
DCA of the nomogram in the training cohort **(A)** and the validation cohort **(B)**. The Y-axis represents a net benefit, and the X-axis represents threshold probability. The green line means no patients died, and the dark green line means all patients died. When the threshold probability is between 0 and 100%, the net benefit of the model exceeds all deaths or none. DCA, decision curve analysis.

**Figure 6:**
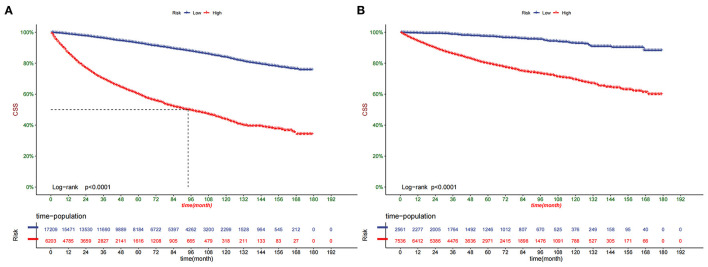
Kaplan-Meier curves of patients in the low-risk and high-risk groups in training cohort **(A)** and validation cohort **(B)**.

**Figure 7:**
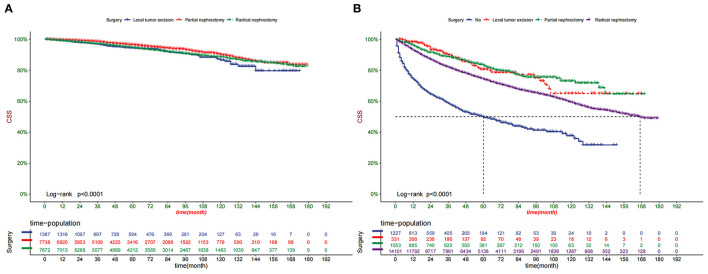
Kaplan-Meier curves of patients with different surgery in the low-risk group **(A)** and high-risk group **(B)**.

Decision curve analysis showed a good clinical application value (Figure 5). Moreover, DCA results showed that the DCA of the nomogram was significantly superior to the traditional TNM staging system in predicting patient survival. We divided the patients into the high-risk group (overall score ≤67.9) and the low-risk group (overall score > 67.9). The K-M curve showed that in the training cohort and validation cohort, the survival rate of patients in the high-risk group was significantly lower than that in the low-risk group (Figure 6). The 1-, 3-, and 5 years survival rates in high-risk group were 0.920 (95% CI, 0.916–0.924), 0.808 (95% CI, 0.802–0.815), and 0.732 (95% CI, 0.724–0.740), respectively. The 1-, 3-, and 5 years survival rates in the low-risk group were 0.992 (95% CI, 0.991–0.994), 0.976 (95% CI, 0.973–0.979), and 0.954 (95% CI, 0.950–0.957), respectively. Based on their risk grouping, we analyzed surgical options for patients in the low-risk and high-risk groups. In the low-risk group, we found that all patients received surgical treatment, and most patients received RN and PN, with significantly higher survival rates than local tumor excision. In the high-risk group, most patients who underwent RN had significantly higher survival rates than those who did not, with the highest survival rates among patients who underwent PN and local tumor excision (Figure 7).

### Online Application of Nomogram

Based on our established programs, we developed a web application to predict 1-, 3-, 5 years CSS in elderly patients with ccRCC. Log on to https://liujiayan.shinyapps.io/DynNomapp/ can enter the website. The survival probability of patients can be obtained by inputting clinicopathological information of patients.

## Discussion

Renal cell carcinoma accounts for about 2% of all cancer diagnoses and deaths worldwide, with a higher incidence in developed countries and a severe burden on economic development. RCC is one of the top ten malignant tumors threatening human health (17). CcRCC is a category of RCC with a poor prognosis (7), and its risk factors are controversial. For example, Faramawi et al. (18) found that red meat consumption was an independent risk factor for the prognosis of ccRCC through meta-analysis, while Eun et al. (19) proved no clear correlation through 13 prospective studies. Similar conclusions also appeared when alcohol consumption (20) and exercise (21) were considered risk factors. Therefore, it is crucial to analyze and screen out specific risk factors, establish a predictive model, and accurately predict the prognosis of ccRCC patients.

Age is a critical factor in the occurrence and development of cancer, and so is ccRCC. With age, the probability of genetic mutations leading to cancer also increases. Aging is associated with highly reproducible DNA methylation changes, which helps to explain the higher prevalence of malignant tumors in the elderly (22). There is no consensus on defining the age of elderly patients, but more than 60% of initial cancer diagnoses and more than 70% of cancer deaths occur in patients over 65 years old (8). To improve the accuracy and representativeness of the prediction model, we included patients with ccRCC over 65 years old in this study. It is worth noting that newly diagnosed cases of ccRCC in the elderly are still mainly manifested by renal masses (8), and these masses usually show low-grade malignancy. However, clinicians mostly choose RN to avoid the poor prognosis caused by tumor metastasis, while such excessive treatment leads to more complications and even death in elderly patients (23, 24) and again highlights the crucial clinical value of this study.

Several studies have shown that married patients have increased cancer relative survival, even for untreated tumors (25–27). It may be related to the emotion of marriage and decision-making of medical treatment. Perini et al. (28) found that depression was a risk factor for the low survival rate of cancer patients, while John et al. (29) found that the incidence of depression in men after marriage was significantly lower than that before marriage, which provided strong evidence for emotional factors. In addition, married cancer patients can detect physical abnormalities early and actively cooperate with treatment, possibly out of the responsibility to the family after marriage (30). In conclusion, married patients showed significant improvement in RR compared with unmarried patients for cancer with a relatively low degree of malignancy, such as patients with ccRCC included in this study. Marriage is not beneficial for highly malignant cancers, such as pancreatic cancer and renal malignant rhabdomyoma tumor (31, 32).

On average, two individuals of the same sex are more than 99.9% alike. However, the genetic similarity between male and female individuals is only 98.5%(33). According to data from the SEER database, men tend to be diagnosed with RCC with higher stage and grade than women (34). Based on data analysis from the Multicenter Society for Renal Oncology Collaborative Study (CORONA) database, women's CSS (hazard ratio [HR] 0.75, *p* < 0.001) and overall survival (OS) (HR 0.80, *p* < 0.001) were significantly better than those of men (35). In this study, the prognosis of female patients with ccRCC was also substantially better than that of male patients (HR: 0.834, 95% CI: 0.796–0.874, *p* < 0.001).

Classical RN involves removing the kidney, perirenal adipose tissue, adrenal gland, and local lymph nodes. It became the preferred treatment for ccRCC for some time. However, the massive loss of nephron caused by surgery can induce chronic kidney disease and cause death (36). Nevertheless, PN can preserve normal nephron to the maximum extent and is widely used in ccRCC cases with tumor sizes <7 cm in length. Nowadays, the biggest challenge for PN is to avoid positive excision margin and reduce postoperative complications, such as hematuria, perirenal hematoma, and urinary fistula (37). Local tumor excision and active monitoring (AS) represented by RFA have been widely used in elderly patients and achieved good results due to contraindicated comorbidity and short-life expectancy. In a meta-analysis of case studies, Dib et al. (38) showed that cryoablation and RFA efficacy were 89 and 90%, respectively. In addition, RFA and other treatments can be operated under the guidance of ultrasound or CT positioning, effectively avoiding the damage of anesthetics to the heart, liver, and other organs of the elderly. Nevertheless, the surgical recurrence rate of local tumor resection is much higher than that of RN and PN (37). This study found that low-risk patients were more likely to benefit from PN and RN, while high-risk patients were more likely to benefit from PN and local tumor excision.

TNM staging of RCC was based on the 2021 European Association of Urology (EAU) updated criteria (39). The Fuhrman grading system was adopted for tumor classification. According to WHO recommendations, grade I was defined as well differentiated, grade II as moderately differentiated, grade III as poorly differentiated, and grade IV as undifferentiated (36). We found that high TNM staging and histologic grade were associated with poor prognosis, which is consistent with the conclusions of previous studies (40).

In recent years, the broad application of the nomogram prediction model has gradually replaced the traditional TNM staging. First, the c-index of traditional TMN staging for prognosis prediction is about 0.6, which is relatively inaccurate. Second, the prognosis of patients is also affected by many non-anatomic factors, such as age, gender, race, marital status, and surgical methods (41). However, the C-index of this study is 0.81, which is of higher accuracy than the traditional TNM system. Nomogram is a data-based graphical computing tool that can estimate the risk of developing a disease based on staging systems, such as the American Joint Commission on Cancer (AJCC) and other key risk factors related to prognosis (42). Today, many kinds of nomograms have been developed and applied to RCC, such as Chen et al. (43), who included 6,105 elderly patients with metastatic RCC in the SEER database from 2010 to 2015, indicating the advantages of PN in elderly mRCC. Michele et al. (44) included 4,541 patients with RCC over 75 years of age in the SEER database from 2004 to 2014 and proved that PN did not increase the mortality of elderly patients. With the update of the SEER database and the improvement of patients' clinical information, this study included 33,509 elderly CCRCC patients from 2004 to 2018, which was superior to previous studies on elderly RCC in terms of the number of cases and c-index accuracy.

Still, there are some limitations to our study. First of all, our study was retrospective, and selection bias was inevitable. Secondly, some variables, such as patients' body mass index (BMI), smoking, drinking, heart disease, and other data cannot be obtained from the SEER database. However, we included significant variables, such as tumor stage, surgery, age, and other vital factors. Finally, our prediction model is only validated internally, and further external validation is necessary to validate the model's accuracy.

## Conclusion

In this study, we explored prognostic factors in elderly patients with ccRCC. We found that age, sex, marriage, TNM stage, surgery, and tumor size were independent risk factors for CSS. We constructed a new nomogram to predict CSS in elderly patients with ccRCC with good accuracy and reliability, providing clinical guidance for patients and physicians.

## Data Availability Statement

The data analyzed in this study was obtained from the Surveillance, Epidemiology, and End Results (SEER) Program, the following licenses/restrictions apply: To request access to the SEER Incidence Databases, you will first need to create a SEER^*^Stat Account and submit a request form. Requests to access these datasets should be directed to: https://service.cancer.gov/seer-data-access. For questions regarding the data access request process, send an email to SEERDataAccess@mail.nih.gov.

## Ethics Statement

Ethical review and approval was not required for the study on human participants in accordance with the local legislation and institutional requirements. Written informed consent from the patients/ participants or patients/participants' legal guardian/next of kin was not required to participate in this study in accordance with the national legislation and the institutional requirements.

## Author Contributions

JW and CZ designed the study. CZ, JW, JL, LJ, ML, and XT collected and analyzed the data. JW drafted the initial manuscript. CZ, TM, JL, ZZ, and DH revised the article critically. CZ, JL, DH, LJ, and XT reviewed and edited the article. All authors approved the final manuscript.

## Funding

This study was supported by the Kunming City Health Science and Technology Talent 1000 training Project [no. 2020-SW (Reserve)-112], Kunming Health and Health Commission Health Research Project (no. 2020-0201-001), Kunming Xishan District Science and Technology Project (no. 2020-Xike word 23), Kunming Medical Joint Project of Yunnan Science and Technology Department (no. 202001 AY070001-271), and Special Key Project of Chongqing Technology Innovation and Application Development (no. Cstc2019jscx-tjsbX0003).

## Conflict of Interest

The authors declare that the research was conducted in the absence of any commercial or financial relationships that could be construed as a potential conflict of interest.

## Publisher's Note

All claims expressed in this article are solely those of the authors and do not necessarily represent those of their affiliated organizations, or those of the publisher, the editors and the reviewers. Any product that may be evaluated in this article, or claim that may be made by its manufacturer, is not guaranteed or endorsed by the publisher.
